# Noncanonical RGS14 structural determinants control hormone-sensitive NPT2A-mediated phosphate transport

**DOI:** 10.1042/BCJ20240122

**Published:** 2025-01-30

**Authors:** W. Bruce Sneddon, Suneela Ramineni, G. Emme Van Doorn, John R. Hepler, Peter A. Friedman

**Affiliations:** 1 Laboratory for GPCR Biology, Departments of Pharmacology and Chemical Biology, University of Pittsburgh School of Medicine, Pittsburgh, PA, U.S.A; 2 Department of Pharmacology and Chemical Biology, Emory University School of Medicine, Atlanta, GA, U.S.A; 3 Structural Biology, University of Pittsburgh School of Medicine, Pittsburgh, PA, U.S.A

**Keywords:** RGS14, phosphorylation, hormone regulation

## Abstract

The sodium phosphate cotransporter-2A (NPT2A) mediates basal and parathyroid hormone (PTH)- and fibroblast growth factor-23 (FGF23)-regulated phosphate transport in proximal tubule cells of the kidney. Both basal and hormone-sensitive transport require sodium hydrogen exchanger regulatory factor-1 (NHERF1), a scaffold protein with tandem PDZ domains, PDZ1 and PDZ2. NPT2A binds to PDZ1. RGS14 persistently represses hormone action by binding to PDZ2. The RGS14 canonical RGS domain, Ras/Rap-binding domains, and G protein regulatory motif cannot explain its regulatory effects on hormone-sensitive phosphate transport because these actions are mediated not only by the PTH receptor, a G protein-coupled receptor (GPCR), but also by the fibroblast growth factor receptor-1, a receptor tyrosine kinase that is not governed by G protein activity. Here, we identify the structural elements of RGS14 that mutually control the action of PTH and FGF23. RGS14 truncation constructs lacking upstream sequence and the RGS domain were fully functional. Removing the linker sequence between the RGS and RBD1 domains abolished RGS14 action. Examination of the α-helical linker region suggested candidate serine residues that might facilitate regulatory activities. RGS14 Ser^266^ and Ser^269^ are phosphorylated in response to PTH and FGF23, and replacement of these residues by Ala eliminated the actions of RGS14 on hormone-sensitive phosphate transport. PTH and FGF23 stimulated the phosphorylation of a peptide construct harboring the sites of purported phosphorylation and full-length RGS14. Mutating Ser^266^Ala and Ser^269^Ala abolished phosphorylation. The results establish that RGS14 regulation of phosphate transport requires targeted phosphorylation within the linker and an intact PDZ ligand.

## Introduction

Parathyroid hormone (PTH) and fibroblast growth factor-23 (FGF23) regulate extracellular phosphate homeostasis by controlling the sequestration of the NPT2A Na-phosphate cotransporter (SLC34A1) from kidney cell membranes. Hormone-triggered NPT2A internalization decreases phosphate absorption and promotes urinary excretion. The regulatory action of PTH [1,2] and FGF23 [3,4] on NPT2A requires the PDZ protein NHERF1 and, as we recently described, is tempered by the G protein signaling regulator-14 (RGS14) 2 [5]. NHERF1 contains tandem PDZ domains. NPT2A binds PDZ1, whereas RGS14 binds to the PDZ2 regulatory domain. Expression of RGS14 in human kidney cells stabilizes the binary [NPT2A:NHERF1] complex, blocking PTH and FGF23 action [5,6].

Twenty canonical mammalian RGS proteins have been described and grouped into four families based on sequence homologies and the presence or absence of included domains [7]. Their distinguishing action, mediated by their shared RGS domains, accelerates GTPase activity by hydrolyzing bound GTP, thereby terminating G protein signaling. RGS14 is a member of the D family [7] (or R12) [8] that includes RGS12 and RGS10. The biological functions of RGS14 are incompletely understood but include tonic suppression of synaptic plasticity and hippocampal-based learning [8–10], reduced myocardial remodeling [11], and altered adipose tissue metabolism [12]. A pioneering GWAS study linked *RGS14* variants to differences in circulating PTH levels [13]. Although the identified SNP was intronic, other coding variants in the PDZ ligand [14] led us to explore whether and how this affected phosphate uptake. Naturally occurring RGS14 Asp^563^Asn, for example, disrupted binding to NHERF1, predictably blocking PTH-sensitive Pi transport [5]. By contrast, the Asp^563^Gly variant bound NHERF1 and supported PTH-inhibitable Pi uptake. PDZ ligand residues are numbered backward from zero at the carboxy terminus. Class I PDZ ligands take the form [Asp/Glu]-[Ser/Thr]-X-Φ, where X is promiscuous and Φ is a hydrophobic residue [15]. The -1 position is considered permissive and, indeed, Ala^565^ variants at this locus were fully tolerated and behaved like wild-type (WT) RGS14, abolishing PTH inhibition of Pi uptake [5]. Notably, mutations that interfered with the binding of RGS14 binding to NHERF1 also hampered RGS14’s capacity to affect PTH-regulated Pi transport. The data highlight the close correlation between the binding of RGS14 to NHERF1 and its ability to restrict PTH-sensitive Pi transport.

RGS14 contains an RGS domain, tandem Ras/Ras binding domains (R1/R2), and a G protein regulatory motif (GPR or GoLoco) [16]. Activated Gα-GTP subunits bind the RGS domain, exerting GAP activity toward heterotrimeric G proteins to terminate GPCR signaling. The tandem RGS14 Ras/Rap-binding domains (RBDs, R1, R2) bind to activated H-Ras-GTP R1 [17,18] and also interact with Rap2-GTP and Raf kinases and Ca^2+^/CaM and CaM-dependent protein kinase (CaMKII) [19,20]. The GPR motif binds to inactive Gαi1/3-GDP, aborting guanine nucleotide exchange (i.e. GDI activity) and anchoring Rgs14 at the plasma membrane [21]. Primates, ruminants, and cetaceans express a 20-amino acid longer, full-length RGS14 protein terminating in a PDZ ligand that is absent in mice, rats, and most other species due to the presence of a UAG stop codon in exon 15 [9]. Another variation on this pattern is present in non-placental metatherian mammals, such as the marsupial opossums and Tasmanian devils, which express a full-length form of Rgs14 but lack a carboxy-terminal PDZ sequence.

Human RGS14, containing a PDZ ligand (-DSAL), binds to NHERF1, whereas rat Rgs14 lacking the PDZ ligand does not. Naturally occurring PDZ ligand variants disrupt the binding of RGS14 to NHERF1, thus abrogating the actions of RGS14 [5]. These findings established the role of RGS14 in controlling PTH- and FGF23-sensitive phosphate transport and underscored the requirement for the RGS14 PDZ ligand in this regulatory activity. Canonical regulatory domains of RGS14, associated with G proteins, cannot explain the effects on hormone-sensitive phosphate transport inasmuch as RGS14 controlled both actions of PTH, mediated by its cognate GPCR, and also those of FGF23 that are transduced by the structurally unrelated receptor tyrosine kinase FGFR1 [4,5]. These observations suggested that the characterized RGS14 domains could not explain the elements of RGS14 that govern its action. We hypothesized that RGS14 regulatory activity requires both an intact PDZ ligand and an upstream element. Based on these considerations, we analyzed the RGS14 structural determinants for the mutual action of PTH and FGF23 action on phosphate transport mediated by NPT2A.

## Results

### Deletion mutants identify the RGS14 region responsible for regulating hormone-sensitive Pi uptake

**Figure 1:**
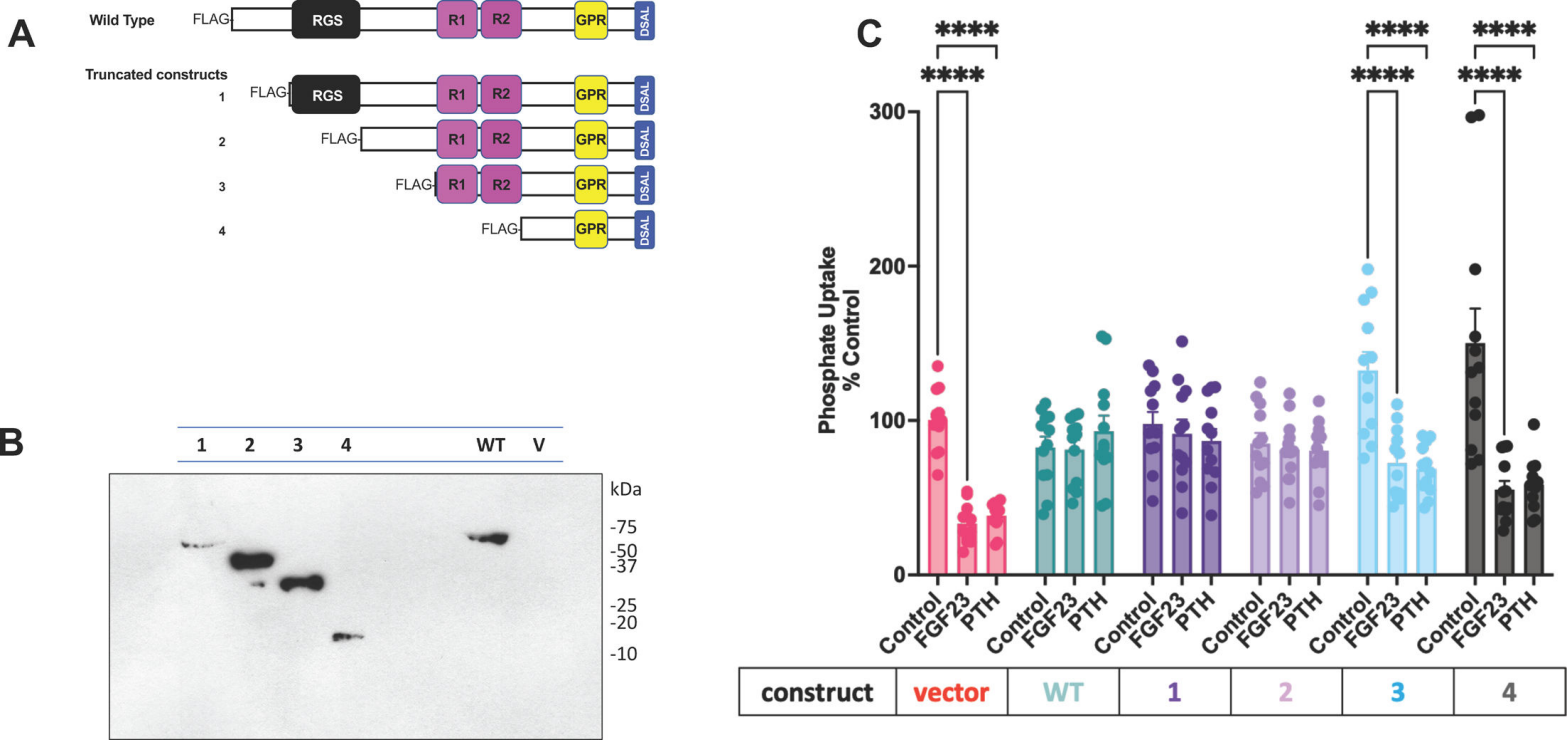
RGS14 blockade of hormone-sensitive phosphate uptake requires the linker region between the RGS and R1 domains. RGS14 truncation mutants were generated to identify functional domains suppressing hormone-regulated phosphate transport. (**A**) Serial deletions from the N-terminus of RGS14 were generated, as outlined in Experimental procedures. Previously characterized functional domains are depicted in the full-length (WT) RGS14 and the truncation mutants (1-**–**4). Regulator of G protein Signaling (RGS) domain, Ras-binding domains 1 and 2 (**R1, R2**), G protein regulatory (Go-Loco) motif, and -DSAL PDZ ligand are depicted. (**B**) Transfected WT-RGS14 and truncation mutants (constructs 1–4) expressed in HEK293 cells. Molecular weight markers (kDa) are presented for reference. Empty vector-transfected cell lysate (V) served as a negative control. (**C**) Hormone-sensitive phosphate uptake was measured in OK cells transfected with WT-RGS14 or the indicated truncation mutant. Phosphate transport was normalized to baseline phosphate uptake under control conditions (100%). Where indicated, cells were treated with 100 nM FGF23 or PTH before phosphate uptake measurements. *n* = 6. ****P < 0.001 *vs*. control.

RGS14 contains an RGS domain, tandem Ras/Ras binding domains (R1/R2), and a G protein regulatory (GPR or GoLoco) motif [6] (Figure 1A). We designed serial truncation mutants starting at the N-terminus to define the RGS14 structural determinants involved in PTH- and FGF23-sensitive phosphate transport. The described functional RGS, R1/R2, domains, and the flanking interlinking regions were progressively deleted (Figure 1A) to ascertain if they were required for RGS14 action on hormone-regulated phosphate transport. FLAG was introduced at the 5′-end of the RGS14 constructs to avoid occluding the 3′-PDZ motif [17]. Truncation construct 1 removed the N-terminal sequence upstream of the RGS domain and retained the remaining protein. Deletion 2 cleaved the N-terminus and RGS domain, leaving the linker region, the downstream R1/R2 domains, and the GPR motif. Further shortening in construct 3 eliminated the linker, while construct 4 removed the two RBD domains, leaving the GPR motif and the C-terminus with its PDZ ligand. Truncation constructs 1–4 were transfected into HEK cells to test their expression. All were detectable by immunoblotting and each conformed to its expected size (Figure 1B). WT-RGS14 was confirmed to be 63 kD. Truncation construct 1, which eliminated the N-terminus up to the RGS domain, ran at 56 kD. Truncation construct 2, in which the N-terminus and the RGS domain were deleted, was detected at 43 kD. Truncation construct 3, which further deleted the linker between the RGS and R1 domains, ran at 30 kD. Truncation construct 4, which removed the N-terminus, RGS, R1 and R2 domains, but left the GPR motif and the PDZ ligand, was detected at 14 kD.

Empty vector, WT, or designated deletion mutants were expressed in OK opossum kidney cells, the widely used model for the action of PTH and FGF23 on phosphate transport [10]. FGF23 and PTH inhibited phosphate uptake in cells transfected with vector (Figure 1C). Transfection with WT RGS14 abolished hormone-sensitive phosphate transport, as reported [5]. Truncation mutants 1 and 2, where the N-terminus or N-terminus plus RGS domain, respectively, were eliminated (Figure 1A), blocking the action of FGF23 and PTH, like full-length RGS14 (Figure 1C), indicating that the critical regulatory regions lie further downstream. In fact, removing the linker region with construct 3 truncated or the linker plus R1/R2 in construct 4 (Figure 1A) abolished the physiological activity of RGS14 on phosphate transport regulated by FGF23 and PTH (Figure 1C). These findings point to a vital function within the linker between the RGS upstream and the R1/R2 downstream domains. Thus, the RGS14 PDZ ligand and an upstream component contained in the putative α-helical linker and not one of the signature RGS14 structural domains are responsible for the regulatory action of RGS14 on hormone-dependent phosphate transport.

### A Ser-rich sequence in the linker between RGS and R1 domains controls hormone action

**Figure 2:**
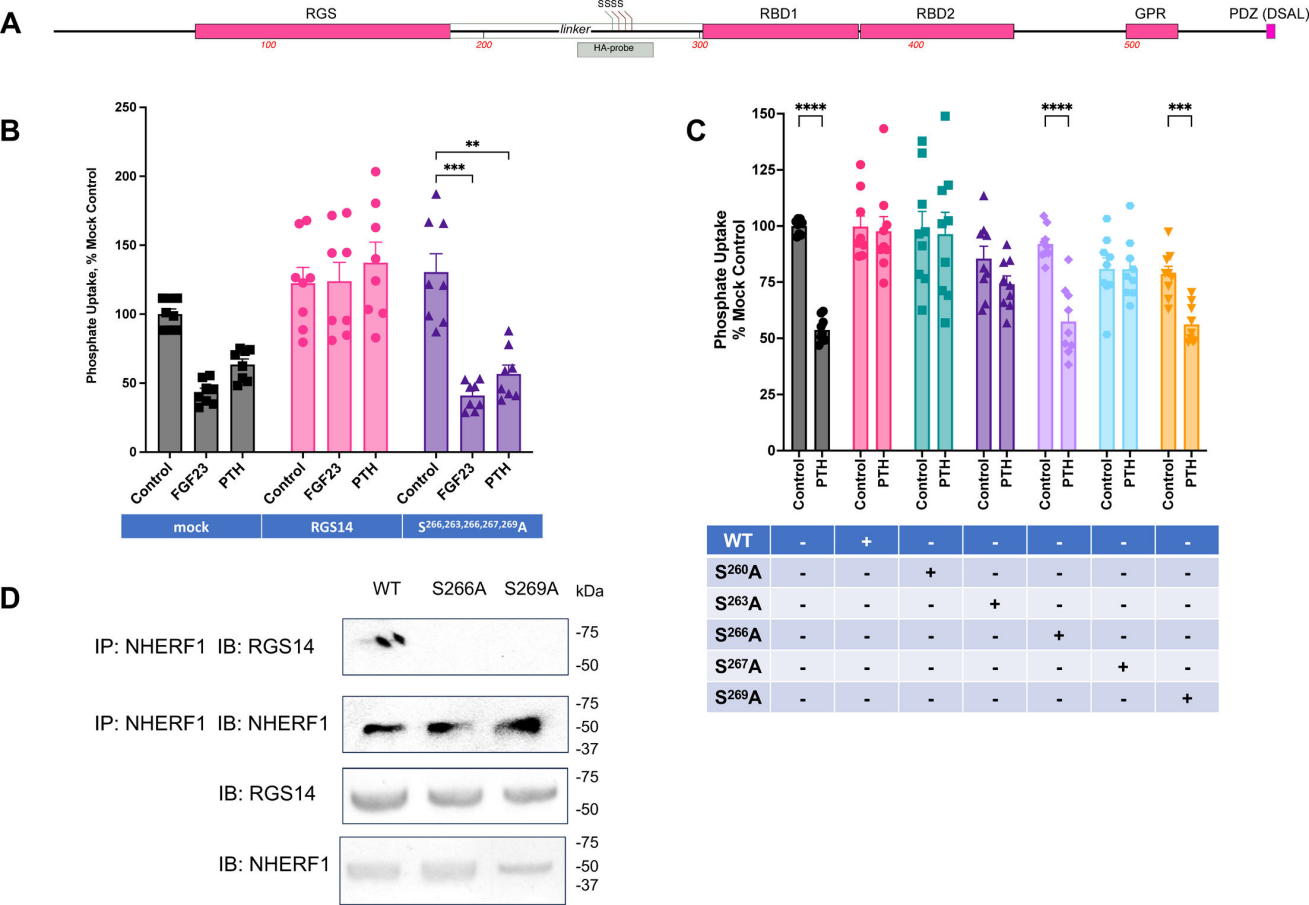
The linker sequence contains specific Ser residues that regulate RGS14 function. (**A**) Schematic RGS14 sequence showing Ser locations in the linker region. (**B**) Combined replacement of Ser with Ala at positions 260, 263, 266, 267, and 269 abolished the ability of RGS14 to regulate hormone inhibition of phosphate transport. As indicated, OK cells were transfected with vector WT-RGS14, or Ser^260, 263, 266, 267, 269^Ala-RGS14. Hormone-sensitive phosphate uptake was measured and normalized to control conditions in vector-transfected cells (100%). *n* = 4. ***P < 0.0001 *vs*. control. **P < 0.001 *vs*. control. (**C**) Substitution of individual Ser with Ala identifies residues in RGS14 controlling hormone-sensitive phosphate transport. OK cells were transfected with empty vector, WT-RGS14, or single Ser/Ala-RGS14 at positions 260, 263, 266, 267, and 269, as indicated. PTH-sensitive phosphate uptake was measured and normalized to control conditions in vector-transfected cells (100%). *n* = 4. ***P < 0.0005 *vs*. control, ****P < 0.0001 *vs*. control. (**D**) Ser^266^Ala and Ser^269^Ala-RGS14 do not immunoprecipitate with NHERF1 OK cells were transfected with HA-NHERF1 and FLAG-RGS14 (WT, Ser^266^Ala, Ser^269^Ala). Forty-eight hours after transfection, lysates were prepared, and NHERF1 was immunoprecipitated using monoclonal anti-HA agarose (Sigma). RGS14 was immunoblotted with a rabbit polyclonal anti-FLAG antibody (Sigma). NHERF1 was immunoblotted utilizing a rabbit polyclonal anti-HA antibody (Sigma). The top two panels represent the immunoprecipitated samples that were analyzed by immunoblot. The bottom two panels are cell lysate samples analyzed by immunoblot. Molecular weight markers are depicted on the right of the blots (kDa), representative of *n* = 4.

We theorized that the targeted phosphorylation of candidate Ser or Thr residues controls the response to hormone challenge. The linker between the RGS and R1 domains contains 18 Ser and 3 Thr residues. P.E.A.R.L, a post-translational modification prediction tool [11], identified a cluster of 5 Ser residues in the linker region as high-probability phosphorylation candidates (Figure 2A). To test the hypothesis that Ser phosphorylation is required for RGS14 function, we initially generated an aggregate construct in which all 5 Ser residues were changed to Ala: Ser^260, 263, 266, 267, 269^Ala. Consistent with this hypothesis, RGS14-Ser^260, 263, 266, 267, 269^Ala abolished PTH and FGF23 inhibition of phosphate uptake (Figure 2B). To determine whether a single Ser residue within this regulatory sequence suffices to govern the functionality of RGS14 in PTH-sensitive phosphate transport, we generated individual Ser-to-Ala mutations at positions 260, 263, 266, 267, and 269. We tested and compared WT RGS14 with each discrete substitution to examine the effect on the inhibition of phosphate transport by PTH. In the absence of RGS14, PTH blocked phosphate uptake (Figure 2C). WT RGS14 abolished the action of PTH, as did the Ser^260^Ala, Ser^263^Ala, and Ser^267^Ala constructs (Figure 2C). In marked contrast, Ser^266^Ala and Ser^269^Ala failed to suppress the modulation of RGS14 of PTH inhibition of phosphate uptake (Figure 2C). These results predict that Ser^266^Ala and Ser^269^Ala should not bind to NHERF1 and, indeed, that was the case (Figure 2D). Taken together, these co-immunoprecipitation findings are consistent with the view that Ser^266^ and Ser^269^ are critical sites for the actions of RGS14 and are compatible with a role for targeted phosphorylation in hormone-mediated inhibition of phosphate transport.

**Figure 3:**
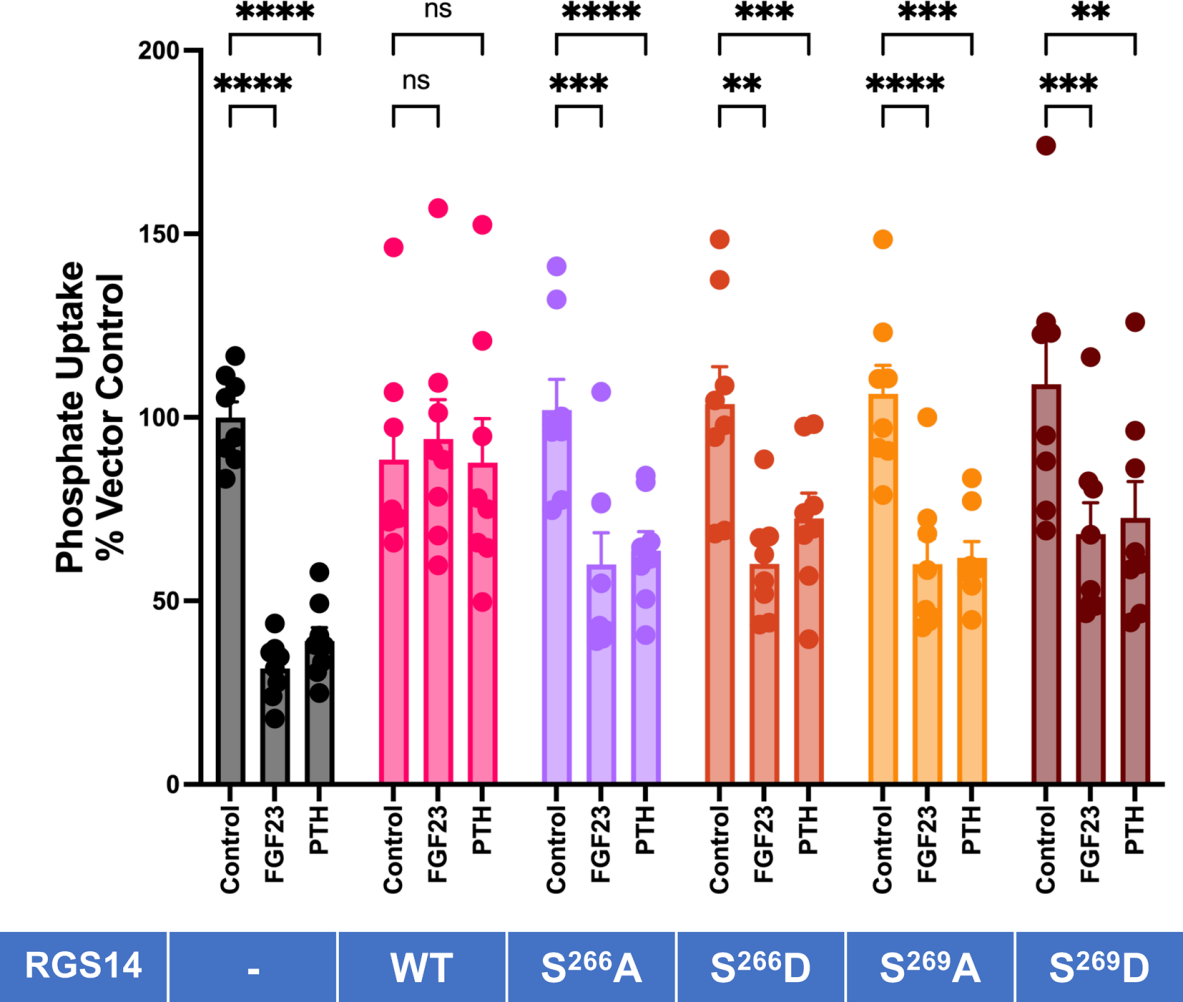
Ser^266^/Ser^269^ mutations interfere with RGS14 actions on PTH/FGF23inhibition of Pi uptake. As indicated, OK cells were seeded on 12-well plates and transfected with RGS14 (WT or mutants) or empty vector (-). Cells were treated with 100 nM FGF23 or PTH for 2 hr, and Pi uptake was measured as detailed in Experimental procedures. Data were normalized to vector control (100%). **P < 0.01 *vs*. control, ***P < 0.001 *vs*. control, ****P < 0.0001 *vs* control. *n* = 4.

We further explored the role of site-specific phosphorylation by generating Ser^266^Asp and Ser^269^Asp phosphomimetics and tested the effect of PTH and FGF23 in side-by-side experiments with the Ser^266^Ala and Ser^269^Ala phosphomutants. The results in Figure 3 demonstrate that phosphomutants and phosphomimetics comparably restored hormone sensitivity to RGS14.

**Figure 4:**
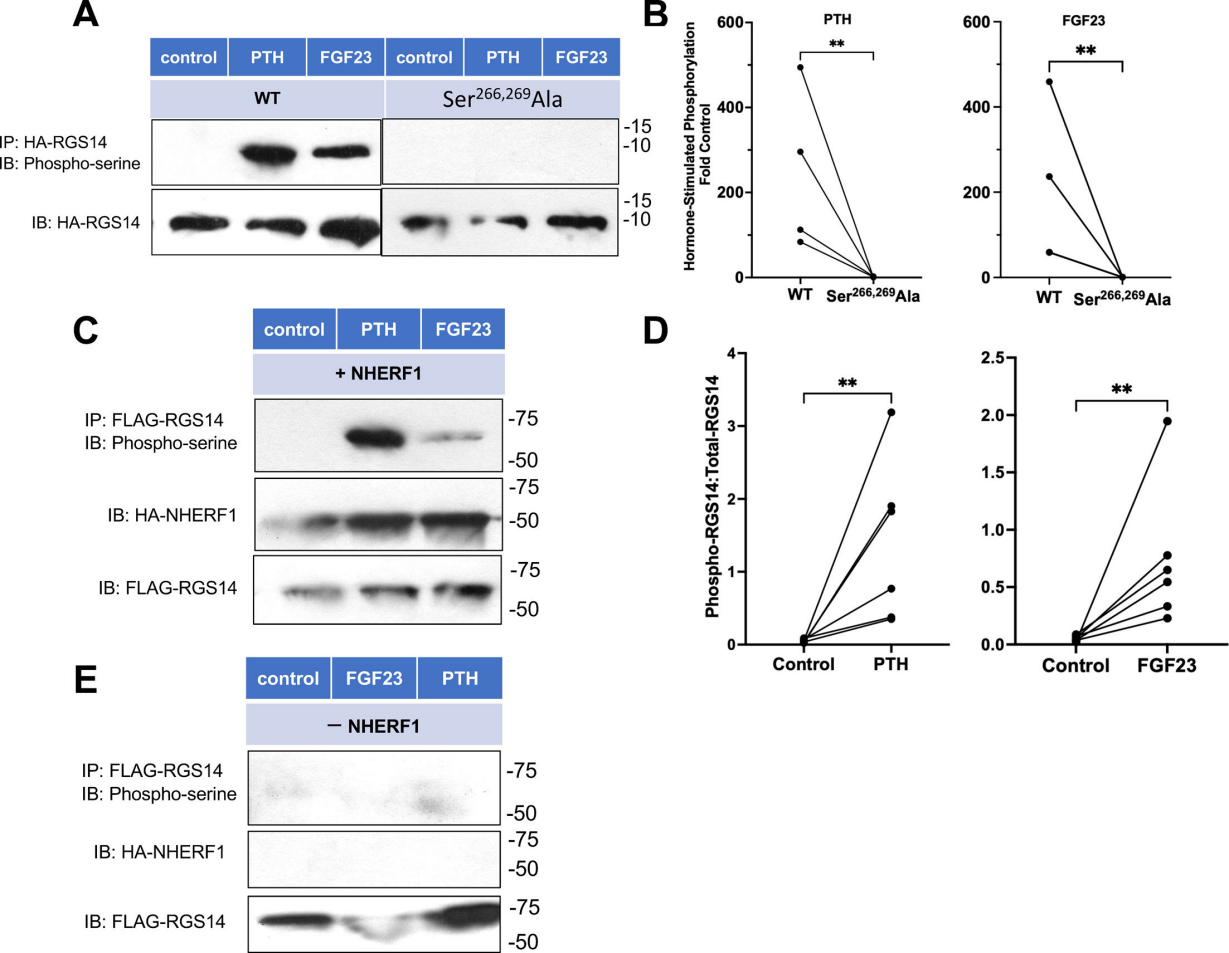
PTH and FGF23 RGS14 phosphorylation. (**A**) RGS14 Ser^266^Ala and Ser^269^Ala phosphorylation. HPCT cells expressing the indicated HA-RGS14 linker were treated with 100 nM PTH or FGF23 for 30 min or vehicle control (**C**). Cell lysates were prepared, and the linker peptide was immunoprecipitated with anti-HA agarose (Methods). The phosphorylated linker was assessed with an anti-phosphoserine antibody. Cell lysates exhibited comparable probe expression. Molecular weight markers (kDa) are shown on the right. Illustrative of *n* = 4. (**B**) Paired analysis of PTH and FGF23 effects on phosphorylation of the wild-typeWT linker peptide or the mutated Ser^266,269^Ala probe. **P < 0.01. (**C**) Hormone-induced phosphorylation of full-length RGS14. HK-2 cells were transfected with FLAG-RGS14 and HA-NHERF1. After 24 h, cells were serum-starved overnight and then treated with 100 nM FGF23 or PTH for 30 min. FLAG-RGS14 was immunoprecipitated as detailed in Experimentalexperimental Proceduresprocedures. Molecular weight markers are shown to the right. Representative of *n* = 6. (**D**) Quantification of hormone-stimulated phosphorylation of full-length RGS14. **P < 0.01. (**E**) PTH and FGF23 failed to promote phosphorylation in HK-2 cells transfected with FLAG-RGS14 but not HA-NHERF1.

Although the mutagenesis experiments are consistent with a presumptive role for Ser phosphorylation, we sought to test directly whether PTH and FGF23 promote targeted phosphorylation. We generated WT and Ser/Ala mutant HA-tagged peptide constructs within the 244-278 RGS14 linker region to accomplish this. The respective linker plasmids were transfected into HPCT cells and, after 24 h, were challenged for 30 min with aqueous vehicle control or 100 nM PTH(1-34) or FGF23. Figure 4A shows that background phosphorylation was undetectable but exposure to PTH and FGF23 elicited robust probe phosphorylation. The Ser^266,269^Ala mutant probe was refractory to PTH and FGF23 (Figure 4A and B). Therefore, RGS14 Ser^266^ and Ser^269^ directly control hormone-regulated phosphate transport.

We extended this examination to corroborate that full-length RGS14 is phosphorylated in response to PTH and FGF23. Here, HK-2 cells were transfected with FLAG-RGS14 and NHERF1. Figure 4C and D illustrate that both PTH and FGF23 stimulated phosphorylation. These experiments provided an opportunity to test the hypothesis that NHERF1 is required for hormone-activated RGS14 phosphorylation. When HK-2 cells were transfected with RGS14 alone, hormone treatment failed to promote RGS14 phosphorylation (Figure 4E).

## Discussion

The objective of the present work was to identify the RGS14 structural elements that mutually control PTH and FGF23 action of PTH. RGS14 governed both PTH and FGF23 actions on phosphate transport. PTH effects are mediated by the cognate PTH1R, a GPCR receptor. FGFR1, an unrelated receptor tyrosine kinase, transmits FGF23 effects on phosphate transport [4]. This observation made it unlikely that RGS14 regulatory activity stemmed from G protein action or one of the integral RGS14 domains associated with G protein function. Accordingly, we initiated the current studies to delineate the RGS14 component responsible for hormone-regulated actions. We employed a strategy involving sequential elimination of the defined regions of RGS14. The results (Figure 1C) disclosed that none of the signature RGS14 domains was responsible for the regulatory activity of phosphate transport. Instead, the findings revealed that the linker region between the RGS and R1 domains is critically involved in hormone-regulated phosphate transport.

We assumed that targeted phosphorylation of one or more of the 21 Ser and Thr residues in the linker was the key required to unlock the regulatory actions of RGS14. There were no evident consensus phosphorylation sites [12] to guide experimental design. However, *in silico* analysis of the region distinguished a high-value sequence, ^260^SQGSLNSSAS^269^, possessing five clustered Ser residues. Initially, all five identified Ser were replaced with Ala. This ensemble approach supported the view that regulation could be attributed to this subset of polar residues.

Further specification with single Ser/Ala substitutions assigned critical roles to Ser^266^ and Ser^269^, which behaved the same as WT RGS14 (Figure 2C). These findings do not exclude the participation of additional Ser or Thr residues within the linker. They also do not speak to possible sequential phosphorylation events. The sequence surrounding Ser^266^ and Ser^269^ is conserved in rodents suggesting that it might serve as a binding motif. Earlier work showed that RGS14 PDZ ligand mutations abrogate binding to NHERF1 and that rat Rgs14 does not bind NHERF1 [5]. Thus, if the conserved region functions as a binding motif, it is not for NHERF1. The absence of a regulatory action of Rgs14 underscores the absolute requirement for the carboxy-terminal PDZ ligand, conspicuously absent in mice and rats.

We expected the Ser^266^Asp and Ser^269^Asp phosphomimetics to show greater constitutive phosphate uptake. However, their action was confined to imparting PTH and FGF23 RGS14 sensitivity, similar to that of the phosphoresistant Ser^266^Ala and Ser^269^Ala mutants. Taken together, these results are consistent with the view that site-specific phosphorylation is necessary for hormone action. The absence of an effect of the Ser^266^Asp and Ser^269^Asp phosphomimetics on baseline action suggests that, in this instance, Asp does not replicate Ser phosphorylation. Phosphomimetic mutagenesis with Asp or Glu introduces an analogous but not necessarily functional identity. The phosphate group has a -2 negative charge compared to the single negative charge of the Asp or Glu carboxylate group. The increased size of the side chain may significantly perturb the local protein structure [22]. We found similar actions at NHERF1 Ser^162^, where Asp failed as a phosphomimetic, but introducing pSer^162^ using amber codon suppression demonstrated an increased affinity for GRK6A [23]. These observations emphasize the limitation of using phosphomimetics to draw conclusions about phosphorylation.

**Figure 5:**
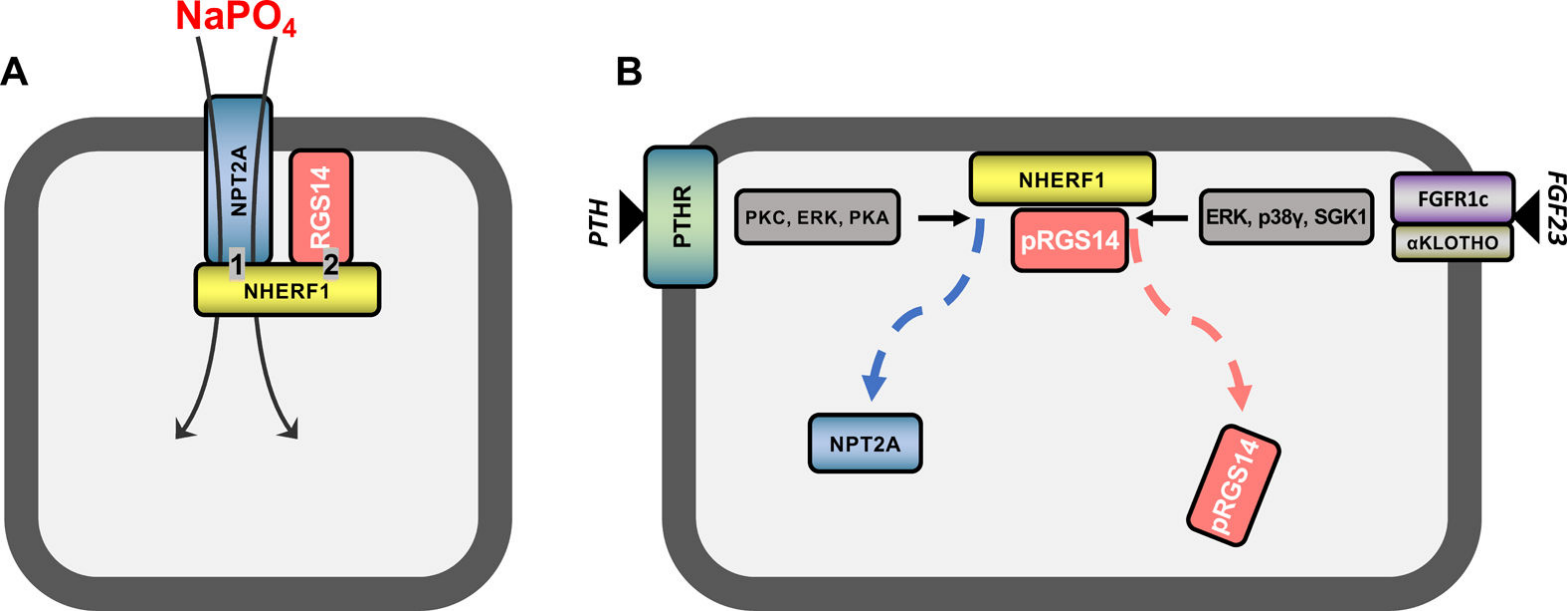
Hormone-responsive phosphate transport. Working model of the role of RGS14 in controlling NPT2A-mediated phosphate uptake under resting conditions (**A**) and following stimulation with PTH or FGF23 (**B**). (**A**) NPT2A binds NHERF1 PDZ1 and human RGS14 binds PDZ2, stabilizing the [NHERF1:NPT2A] complex at the membrane surface permitting constitutive phosphate uptake. (**B**) PTH, acting through the PTH receptor (PTHR) or FGF23 through FGFR1, promotes RGS14 phosphorylation and its dissociation from NHERF1 leading to sequestration of NPT2A and cessation of phosphate uptake [5,6].

The PTHR and FGFR1 signaling pathways impinging on RGS14 remain to be defined. Earlier work disclosed that PTH and FGF23 exert shared actions on phosphate transport in some instances, while their effects are singular and discrete in others. PTH and FGF23 share select phosphorylation sites on NHERF1 [14]. It is not yet known whether a mutually activated kinase is responsible for Ser phosphorylation of the RGS14 linker. The linker contains a dibasic PKA consensus sequence (RRXS/T) that includes Ser^260^. Neither Ser^266^ nor Ser^269^, which eliminated RGS14 activity, are part of a conserved or degenerate consensus PKA phosphorylation motif.

RGS14 has been reported to be highly phosphorylated [14], and considerable evidence indicates that its phosphorylation state regulates its actions. Rat Rgs14 is phosphorylated by PKA at Thr^494^ outside the linker region to regulate Gαi1-GDP interactions [22]. Within the linker region, Rgs14 is phosphorylated at Ser^218^ to regulate 14-3-3γ binding and nuclear localization [24]. Consistent with these findings, we show here that phosphorylation of RGS14 at either Ser^266^ or Ser^269^ regulates its actions toward hormone-sensitive phosphate uptake in kidney cells. A model incorporating these findings and our current grasp of the protein**–**protein interactions governing hormone-responsive phosphate transport mediated by NPT2A are depicted in Figure 5. Additional work beyond the scope of the present investigation will be needed to define the kinases and phosphatases responsible for Ser phospho cycling and to search for other candidate Ser or Thr residues involved. Particularly important will be to define additional participants coordinating this biological pas de deux.

We conclude that RGS14 contains two structural elements that control basal and hormone-regulated phosphate transport. The first is the canonical carboxy-terminal PDZ ligand, which facilitates binding of RGS14 to NHERF1 PDZ2 [5]. Second, we show here that the RGS14 linker is critical and contributes to the regulatory control of PTH- and FGF23-sensitive phosphate transport mediated by NPT2A.

## Experimental procedures

### Chemical reagents, plasmids, and antibodies

Antisera, their respective dilutions, and sources are provided in Table 1 . [Nle^8,18^,Tyr^34^]PTH(1–34) was from Bachem, Torrance, CA (H9110). Recombinant human Arg^179^Gln-FGF23^25-251^ (herein referred to as FGF23), which is resistant to furin cleavage and inactivation, was obtained from R&D Systems (2604-FG-025).

**Table 1:** Antisera, dilutions, sources

Antiserum	Dilution	Source	Catalogue number
Monoclonal anti-HA agarose	1:10 – 1:50	Sigma	A2095
Rabbit polyclonal anti-FLAG	1:1000	Sigma	F7425
Rabbit polyclonal anti-HA	1:1000	Santa Cruz	sc-805
Protein G + agarose	1:10 – 1:100	Santa Cruz	sc-2002
Rabbit polyclonal anti-RGS14	1:1000	Proteintech	16258–1-AP
Anti-human-NPT2A	1:1000	Novus Biologicals	NBP242216
Anti-actin	1:1000	Bachem	H9110
Anti-phosphoserine-HRP	1:10,000	Rockland/Fisher	600–403-261

### Human RGS14 constructs

FLAG-tagged deletion mutants of RGS14 were created using the full-length human RGS14-pcDNA3.1 as a template for PCR using primers listed in Table 2. All PCR products were digested and cloned into pcDNA3.1 + between the HindIII and EcoRI restriction sites.

**Table 2:** Primers used to generate RGS1

Primer	Primer sequence
Common reverse	TGTGCTGGATATCTGCAGAATTCTCAGAGGGCTGAGTCGGTGGTGGAGTTCA
Truncation mutant 1 forward	CTAGCGTTTAAACTTGCCACCATGGACTACAAAGACGATGACGATAATCCTTCGAGCGGCTGTTGCAGGACC
Truncation mutant 2 forward	CTAGCGTTTAAACTTAAGCTTGCCACCATGGACTACAAAGACGATGACGATAAACTAGCCGAAGCCGAGGGACGCCCTC
Truncation mutant 3 forward	CTAGCGTTTAAACTTAAGCTTGCCACCATGGCTACAAAGACGATGACGATAAAAAGTACTGCTGTGTGTACCTGCCC
Truncation mutant 4 forward	CTAGCGTTTAAACTTAAGCTTGCCACCACCATGGACTACAAAGACGATGACGATAAACTTCCAGGTGAAGATCTCCAAAG

Single Ser-to-Ala substitutions were introduced into FLAG-RGS14 using the Agilent QuikChange Lightning Site-Directed Mutagenesis Kit (210519) and primers detailed in Table 3.

**Table 3:** Primers used to generate single RGS14 Ser-Ala mutations in the linker region

Forward primer	Reverse primer
S260A GCCTTGCGCCGAGAGGCTCAGGGCTCCCTCAAC	GTTGAGGAGCCCTGAGCCTCTCGGCGCAAGGC
S263A CGAGAGTCTCAGGGCGCCCTCAACTCCTCCGCC	GGCGGAGGAGTTGAGGGCGCCCTGAGACTCTCG
S263A CGAGAGTCTCAGGGCGCCCTCAACTCCTCCGCC	GTCCAGGCTGGCGGAGGCGTTGAGGGAGCCCTG
S267A CAGGGCTCCCTCAACTCCGCCGCCAGCCTGGACCTTGGC	GCCAAGGTCCAGGCTGGCGGCGGAGTTGAGGGAGCCCTG
S269A CCCTCAACTCCTCCGCCGCCCTGGACCTTGGTTC	GAAGCCAAGGTCCAGGGCGGCGGAGGAGTTGAGGG

To examine the direct phosphorylation of the RGS14 linker region and the role of Ser^266^/Ser^269^, we designed two probes and commissioned their synthesis by a commercial source (VectorBuilder, Chicago, IL). The WT probe consisted of an HA-tag upstream of the RGS14 linker sequence: ATGTCAGATGGATCCATGTATCCGTATGATGTGCCGGATTATGCGGCGAACGCGGCGCTGCGCCGCGAAAGCCAGGGCAGCCTGAACAGCAGCGCGAGCCTGGATCTGGGCTTTCTGGCGTTTGTGAGCAGCTGAGAATTCGTCTAG. The modified Ser^266,269^Ala probe consisted of the HA-tag upstream of a mutated RGS14 linker sequence: ATGTATCCGTATGATGTGCCGGATTATGCGGCGA-ACGCGGCGCTGCGCCGCGAAAGCCAGGGCAGCCTGAACGCCAGCGCGGCCCTGGATCTGGGCTTTCTGGCGTTTGTGAGCAGCTGAGAATTCGTCTAG. VectorBuilder synthesized these probes in a proprietary vector. The map is provided in Figure S1.

### Cell lines and culture

Opossum kidney cells (OK/B) were obtained from J. Cole [25] and cultured in DMEM/F-12 (Corning, 10-090-CV) supplemented with 5% heat-inactivated fetal bovine serum (GenClone 25-514H, Genesee Scientific) plus 1% penicillin and streptomycin.

Human Proximal Convoluted Tubule cells (HPCT-05-wt cells, hereafter HPCT) were obtained through Dr. Ulrich Hopfer. HPCT cells were propagated in DMEM/F12 (Corning, 10-092-CV) supplemented with 10 ng/ml recombinant human epidermal growth factor, 3.5 μg/ml ascorbic acid, 1 mg/mL insulin, 0.55 mg/mL transferrin, 0.5 μg/ml sodium selenite, 25 ng/ml hydrocortisone, 5% heat-inactivated fetal bovine serum (GenClone 25-514H, Genesee Scientific), plus 1% penicillin and streptomycin.

HK-2 human proximal kidney cells [26] were cultured in DMEM/F12 (Corning, 10-092-CV) supplemented with 5% heat-inactivated fetal bovine serum (GenClone 25-514H, Genesee Scientific) plus 1% penicillin and streptomycin.

Human Embryonic Kidney (HEK293) cells were cultured in DMEM (Corning, 10-090-CV) supplemented with 10% heat-inactivated fetal bovine serum (GenClone 25-514H, Genesee Scientific) plus 1% penicillin and streptomycin.

### Pi transport

OK cells were seeded on 12-well plates. Twenty-four hours later, as indicated, cells were transfected with 1 µg/well of WT or mutant FLAG-RGS14 plasmid, using Lipofectamine 3000 (Invitrogen). After 48 h, cells were serum-starved overnight and then treated for 2 h with 100 nM PTH(1-34) or FGF23, as indicated. Pi uptake was measured as described [4]. The hormone-supplemented medium was aspirated, and the wells were washed three times with 1 mL of Na-replete wash buffer (140 mM NaCl, 4.8 mM KCl, 1.2 mM MgSO_4_, 0.1 mM KH_2_PO_4_, 10 mM HEPES, pH 7.4). The cells were incubated with 1 µCi [^32^P]orthophosphate (PerkinElmer Life Sciences, NEX053) in 1 mL of Na-replete buffer for 10 min. Pi uptake was stopped by placing the plate on ice and rinsing the cells three times with Na-free buffer (140 mM N-methyl-D-glucamine, 4.8 mM KCl, 1.2 mM MgSO_4_, 0.1 mM KH_2_PO_4_, 10 mM HEPES, pH 7.4). The cells in each well were extracted overnight at 4°C using 500 μl 1% Triton X-100 (Sigma). A 250 μl aliquot was counted in a Beckmann Coulter LS6500 scintillation spectrometer. Data were normalized to Pi uptake under control conditions defined as 100%.

### Transfection and immunoblotting

Protein lysates were prepared from OK and HEK293 cells transfected with the indicated RGS14 variants. Cells were seeded on 6-well plates. Twenty-four hours later, the cells were transfected with 1 μg/well of human RGS14 expression plasmid using Lipofectamine 3000 (Invitrogen). After 48 h, protein lysates were prepared using 1% Nonidet P-40 buffer supplemented with protease inhibitor mixture I.

Cells (OK/B, HEK293) were seeded on 6-well plates concurrently with the 12-well plates used in the phosphate uptake measurements. After 24 h, cells were transfected with 1 µg each/well of HA-NHERF1 and/or FLAG-RGS14 (WT, mutants as indicated) using Lipofectamine 3000 (Invitrogen). Forty-eight hours post-transfection, cells were lysed with 1% Nonidet P-40 (50 mM Tris, 150 mM NaCl, 5 mM EDTA, 1% Nonidet P-40) supplemented with protease inhibitor mixture I (EMD Millipore). Lysis was performed for 15 min on ice, and membrane components were pelleted at 12000 rpm for 15 min at 4°C in an Eppendorf 5415 R refrigerated microcentrifuge. The supernatant was mixed with an equal volume of SDS–PAGE sample buffer (Bio-Rad) supplemented with 5% β-mercaptoethanol (Sigma). These protein samples were resolved on 10% SDS–polyacrylamide gels and transferred to Immobilon-P membranes (Millipore) using the semidry method (Bio-Rad). Membranes were blocked for 1 h at room temperature with 5% nonfat dried milk in Tris-buffered saline plus Tween 20 (TBST) (blocking buffer) and incubated with the indicated primary antibodies at a dilution of 1:1000 in blocking buffer overnight at 4°C. The membranes were washed four times for 10 min in TBST and then incubated with the secondary antibodies (goat anti-rabbit IgG or donkey anti-mouse IgG conjugated to horseradish peroxidase) at a 1:5000 dilution for 1 h at room temperature. Membranes were washed four times for 10 min in TBST. Protein bands were detected by luminol-based enhanced chemiluminescence (EMD Millipore WBKLS0500). Immunoblots were captured on film and quantified using Image J.

### Phosphorylation of the RGS14 linker region

HPCT cells were seeded on 6-well plates and transfected with HA-tagged WT- or Ser^266,269^Ala-RGS14 linker expression plasmids. Twenty-four hours later, cells were serum starved overnight and then treated for 30 min with 100 nM PTH(1-34) or FGF23, as indicated. Cell lysates were prepared and incubated with agarose-conjugated monoclonal anti-HA beads overnight at 4°C at a dilution of 1:50. Immunoprecipitated protein was washed four times in NP40 buffer and analyzed by immunoblot using an HRP-conjugated rabbit polyclonal anti-phosphoserine antibody (1:10,000 dilution). As a control for immunoprecipitation, duplicate blots were analyzed by immunoblot using a rabbit polyclonal anti-HA antibody (1:1000 dilution) and a donkey anti-rabbit HRP conjugated secondary antibody (1:5000 dilution). Protein bands were detected by luminol-based enhanced chemiluminescence (EMD Millipore WBKLS0500).

The extent of phosphorylation was quantified by scanning densitometry using Image J. Bands representing phosphorylated peptides were normalized by dividing by the density of the respective total lysate. Hormone-stimulated phosphorylation was calculated by dividing the normalized PTH/FGF23 densitometry by the normalized control densitometry.

### Phosphorylation of Full-Length RGS14

HK-2 cells were seeded on 6-well plates and transfected with FLAG-RGS14-pcDNA3.1 with or without HA-NHERF1-pcDNA3.1. 24 hr later, cells were serum starved overnight and then treated for 30 min with 100 nM PTH(1-34) or FGF23. Cell lysates were prepared and incubated with agarose-conjugated monoclonal anti-FLAG beads overnight at 4°C at a dilution of 1:50. Immunoprecipitated protein was washed 4 times in NP40 buffer and analyzed by immunoblot using an HRP-conjugated rabbit polyclonal anti-phosphoserine antibody (1:10,000 dilution). As a control for immunoprecipitation, duplicate blots were analyzed by immunoblot using a rabbit polyclonal anti-HA antibody (1:1000 dilution) (for NHERF1 in lysates) or a rabbit polyclonal anti-FLAG antibody (1;1000 dilution) (for RGS14 in lysates) and a donkey anti-rabbit HRP conjugated secondary antibody (1:5000 dilution). Protein bands were detected by luminol-based enhanced chemiluminescence (EMD Millipore WBKLS0500). Phosphorylation was quantified by scanning densitometry using Image J. Bands representing phosphorylated immunoprecipitated FLAG-RGS14 were normalized by dividing by the density of the respective total lysate sample (phospho-RGS14:total RGS14).

### Statistical analysis

Results were analyzed using Prism 10 software (GraphPad, La Jolla, CA). Data represent the mean ± SD or SEM as indicated by *n* ≥ 3 independent experiments and were compared by analysis of variance with *post hoc* testing using the Bonferonni procedure or paired t-test as appropriate. Values of *P* < 0.05 were considered statistically significant.

## Supplementary material

online supplementary figure 1.

online supplementary material 1.

## Data Availability

A. A detailed methods section will be provided outlining the data collection generated with this work. Any step-by-step protocols developed in this project will be shared as a supplementary protocol document. Specifications about instruments and technologies used to produce these data are included. B. Original data are archived on Open Science Framework (OSF). C. All reagents, such as novel RGS14 constructs and techniques and detailed methods, will be made available upon written request once published. The institution and PIs adhere to the NIH Grants Policy on Sharing Unique Research Resources, including Sharing Biomedical Research Resources: Guidelines for Recipients of NIH Grants and Contracts on Obtaining and Disseminating Biomedical Research Resources.

## Author note

Human proteins are indicated by three-letter uppercase convention; genes in italics. Only the first letter is uppercase for the corresponding mouse protein or gene.

## Abbreviations

FGF23: fibroblast growth factor-23
GAP: GTPase activating protein
GPR: G protein regulatory motif
HEK293: Human Embryonic Kidney 293 cells
HK-2 cells: human kidney-2 cells
HPCT cells: human proximal convoluted tubule cells
NHERF1: sodium-hydrogen exchange factor-1
NPT2A: sodium-phosphate cotransport protein 2A
PDZ: postsynaptic density protein 95 (PSD95), drosophila disc large tumor suppressor (DlgA), and zonula occludens 1 protein (ZO1)
PTH: parathyroid hormone
PTHR: PTH receptor
RBD: Ras-binding domain
RGS14: regulator of G protein signaling 14
