# Supplementary material for: Noncanonical RGS14 structural determinants control hormone-sensitive NPT2A-mediated phosphate transport
Source: Biochem J. 2025 Jan 30;482(3):BCJ20240122. doi: 10.1042/BCJ20240122 (PMC12133299; doi:10.1042/BCJ20240122)
Supplement: online supplementary figure 1. [file bcj-482-3-BCJ20240122-s001.docx]

**
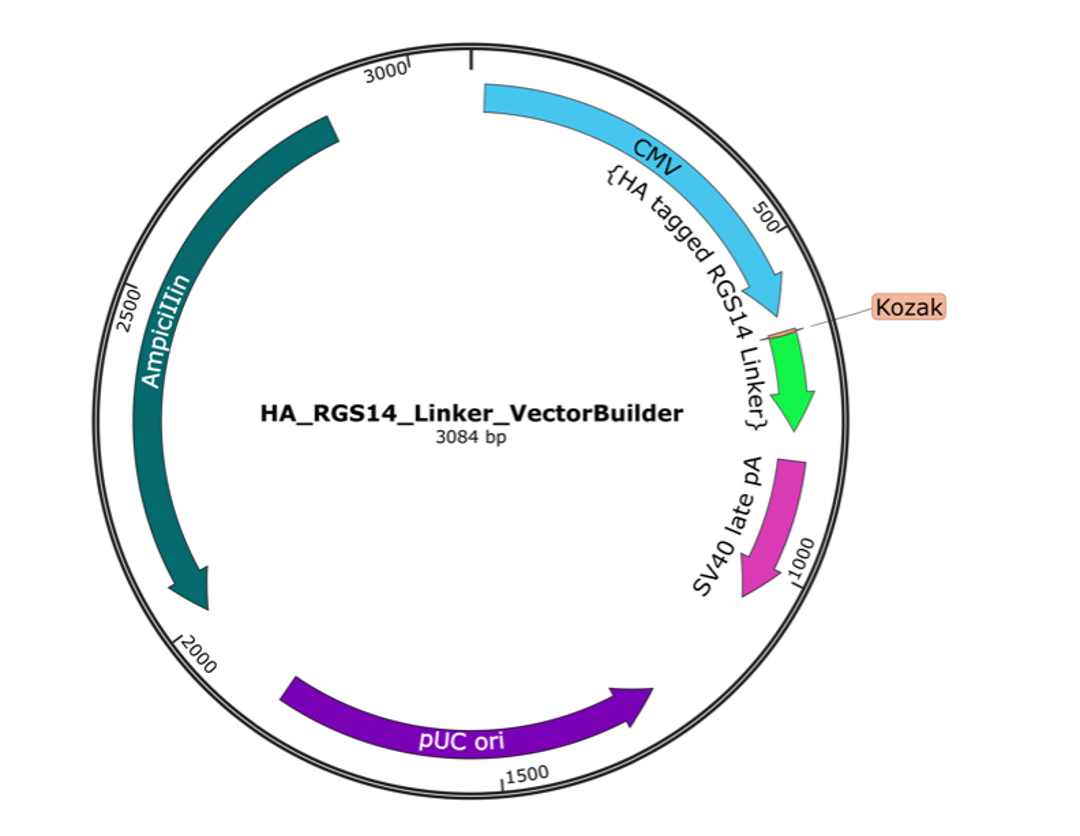
**

**Figure S1: HA-RGS14 Linker Expression Plasmid.**

Wild-type and Ser^266,269^Ala mutant HA-tagged peptide constructs within the RGS14 244:278 linker region. Constructs were commercially synthesized by VectorBuilder (Chicago, IL). The peptide sequences used in this vector are listed in Methods.
